# Bothnian Palmoplantar Keratoderma: Further Delineation of the Associated Phenotype

**DOI:** 10.3390/genes13122360

**Published:** 2022-12-14

**Authors:** Laura Fertitta, Fabienne Charbit-Henrion, Stéphanie Leclerc-Mercier, Thao Nguyen-Khoa, Robert Baran, Caroline Alby, Julie Steffann, Isabelle Sermet-Gaudelus, Smail Hadj-Rabia

**Affiliations:** 1 Department of Dermatology, Hôpital Necker-Enfants Malades, AP-HP Centre Université Paris Cité, 149 rue de Sèvres—75743 PARIS, CEDEX 15, 75679 Paris, France; 2 Reference Center for Genodermatoses and Rare Skin Diseases (MAGEC), Hôpital Necker-Enfants Malades, AP-HP Centre Université Paris Cité, 75015 Paris, France; 3 INSERM U1163, Institut Imagine, Hôpital Universitaire Necker-Enfants Malades, 75015 Paris, France; 4 Genomic Medecine Unit, Hôpital Necker-Enfants Malades, AP-HP Centre Université Paris Cité, 75015 Paris, France; 5 Department of Pathology, Hôpital Necker-Enfants Malades, AP-HP Centre Université Paris Cité, 75015 Paris, France; 6 Laboratory of Biochemistry, Hôpital Necker-Enfants Malades, AP-HP Centre Université Paris Cité, 75015 Paris, France; 7 Reference Center for Rare Diseases: Cystic Fibrosis and Other Epithelial Respiratory Protein Misfolding Diseases, Hôpital Necker-Enfants Malades, AP-HP Centre Université Paris Cité, 75015 Paris, France; 8 INSERM U1151, Institut Necker-Enfants Malades, Université Paris Cité, 75015 Paris, France; 9 Nail Disease Center, 06400 Cannes, France

**Keywords:** palmoplantar keratoderma, genodermatosis, aquaporin 5, sweat test

## Abstract

Bothnian palmoplantar keratoderma (PPKB, MIM600231) is an autosomal dominant form of diffuse non-epidermolytic PPK characterized by spontaneous yellowish-white PPK associated with a spongy appearance after water-immersion. It is due to *AQP5* heterozygous mutations. We report four patients carrying a novel *AQP5* heterozygous mutation (c.125T>A; p.(Ile42Asn)), and belonging to the same French family. Early palmoplantar swelling (before one year of age), pruritus and hyperhidrosis were constant. The PPK was finally characterized as transgrediens, non-progrediens, diffuse PPK with a clear delineation between normal and affected skin. The cutaneous modifications at water-immersion test, “hand-in-the-bucket sign”, were significantly evident after 3 to 6 min of immersion in the children and father, respectively. AQP5 protein is expressed in eccrine sweat glands (ESG), salivary and airway submucosal glands. In PPKB, gain of function mutations seem to widen the channel diameter of ESG and increase water movement. Thus, swelling seems to be induced by hypotonicity with water entrance into cells, while hyperhidrosis is the result of an increased cytosolic calcium concentration.

## 1. Introduction

Palmoplantar keratodermas (PPK) represent a diverse group of hereditary and acquired disorders characterized by hyperkeratosis of the skin of the palms and soles. Among the hereditary PPK, the Bothnian type (PPKB, MIM600231) is a rare autosomal dominant form of diffuse non-epidermolytic PPK related to heterozygous mutations in aquaporin 5 (*AQP5*). Its prevalence varies from 0.3 to 0.55% in the two northernmost provinces of Sweden [1] and remains unknown elsewhere.

PPKB is characterized by a homogeneous and diffuse PPK ranging from barely detectable to prominent, with a distinct and sometimes papular border. The affected skin often has a yellowish tint. A spontaneous whitish and spongy appearance after water-immersion seems to be the first manifestation, appearing after one year of age [1]. Secondary fungal infections seem to be frequent [1,2,3,4,5].

Moreover, the whitish and spongy appearance after water immersion may mimic acquired aquagenic PPK such as hereditary papulotranslucent acrokeratoderma (HPA) [6] and aquagenic wrinkling of the palms (AWP) [7]. In both, translucent papules on palms and soles (HPA), or palms only (AWP), are induced by water immersion of normal skin. AWP has been associated with mutation(s) in the *CFTR* gene [8,9].

To delineate and better characterize the PPKB phenotype, we report four patients from the same French family, carrying a novel *AQP5* heterozygous mutation.

## 2. Patients and Methods

### 2.1. Clinical and Molecular Characterization

Patients were referred to the outpatient consultation of the Department of Dermatology for aquagenic PPK. Clinical examination was performed by two experienced dermatologists (LF and SHR). It included ultraviolet (UV) light examination (Woods Lamp). A water immersion test, consisting of an immersion of the right palm and sole in warm water (37 °C), with regular acquisition of photographs (before immersion, and after 3 and 6 min), was performed. A nail examination, evaluated against standardized photographs, was performed by an expert dermatologist (RB).

Swabs of the palms and soles were collected for bacteriological and fungal analysis. A skin biopsy was taken from the axillae of patient III-2 for standard characterization.

After written informed consent, DNA was extracted from the blood leucocytes and molecular analyses performed by next-generation sequencing (NGS) using a panel of 150 genes involved in genodermatoses including all genes of inherited PPK in all affected relatives, the mother (III-1) and an unaffected child (IV-3).

### 2.2. Sugar and Sweat Tests

Evaluation of the saliva secretion rate using a 5.95 g sugar cube was performed [10]. A complete dissolution at 4 min or less was considered as normal.

A sweat chloride (Cl^−^) concentration test (SCT) was performed using a Macroduct Advanced 3710 device (www.elitechgroup.com) for pilocarpine iontophoresis, a Macroduct for sweat collection, and a chloridometer 926S (Servilab, Le Mans, France). A Cl^−^ concentration in the sweat greater than 60 mmol/L is deemed to be characteristic of cystic fibrosis (CF) [11]. Normal SCT values were <30 mmol/L, for values between 30 and 59 mmol/L, a CF diagnosis could not be excluded [11].

## 3. Results

### 3.1. Clinical and Molecular Characterization

**Figure 1:**
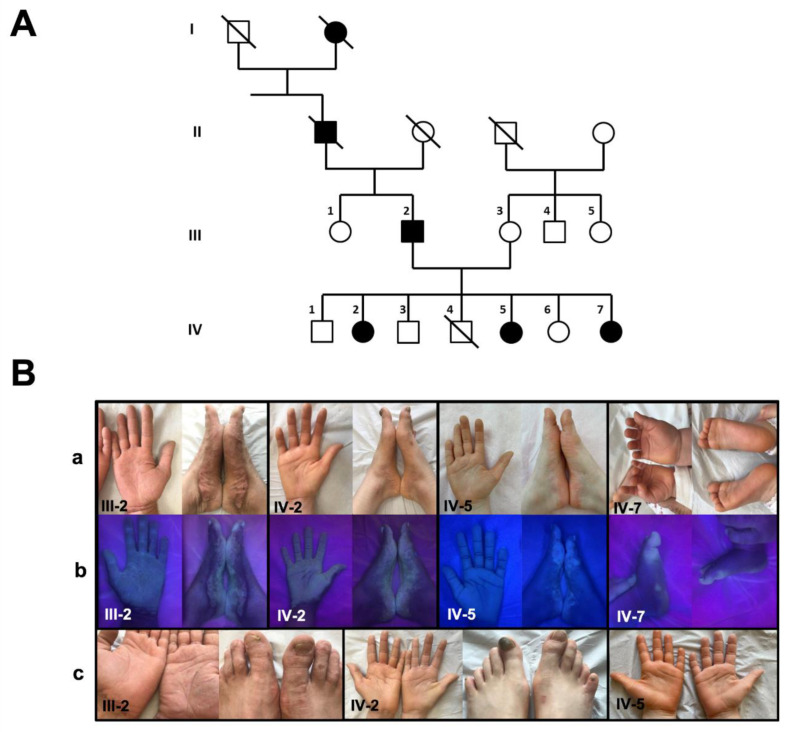
**PPKB: Pedigree and clinical phenotype (present report).** (**A**) Pedigree of the family shows that the clinical phenotype co-segregates with *AQP5* variants; (**B**) clinical pictures of patients III-2, IV-2 and IV-7 (a), enhanced by UV light (b) and after 6 min of water-immersion of right feet and hands (c) for patients III-2 and IV-2. Square = male; round = female; full = affected; slash = deceased.

Since birth, the proband (IV-2, Figure 1A) presented with palmoplantar swelling occurring a few minutes after water immersion.

Her two sisters (patients IV-5 (5-year-old), and IV-7 (11-month-old)) and their 43-year-old father (patient III-2) presented with similar manifestations (Figure 1B). The palmoplantar swelling after water immersion was reported from birth for patients III-2, IV-2 and IV-5, and from the age of 6 months for patient IV-7. All four patients mentioned recurrent pruritus boosted by water-immersion and significant palmoplantar hyperhidrosis with unpleasant odor that interferes with interpersonal relations. Hyperhidrosis, PPK thickness and a spongiotic aspect of the skin worsened during the summer for patient III-2. While swelling was less intense in patient IV-7, it worsened and extended with age in patient III-2. No heat intolerance or pain was observed in patients III-2, IV-2 and IV-5. Dermatological examination showed a major, homogeneous and diffuse yellowish PPK with a distinct papular, inflammatory, pigmented border (patient III-2, Figure 1B), and a focal and slightly thickened predominance on the weight-bearing areas of the foot (patient IV-7). Patients IV-2 and IV-5 presented with similar homogeneous and diffuse yellowish PPK without a papular, inflammatory border. In all patients, the transgrediens and non-progrediens PPK includes toes and fingers. Recent palmoplantar blisters were reported in patient IV-5. Woods light highlighted the affected palmoplantar areas (Figure 1B). Nails were normal in all patients but patient IV-2 (Beau’s lines of the first left toenail, Figure 1B). Neurological examination, including the evaluation of mechanical and thermal sensitivity, was normal in all patients. Patients did not complain of xerostomia. Family history was free from pulmonary manifestations.

After 3 min of the water immersion test, white papules and a whitish spongy appearance were noted on the right (immersed) palm and sole compared to the left (control) palm and sole in all patients. They were more visible at 6 min, particularly in patient III-2 (Figure 1B).

Microbiological swabs were positive for *Staphylococcus aureus* (patient III-2), a Gram-positive bacillus of the commensal genus *Corynebacterium* and a coagulase-negative *Staphylococcus* (patient IV-2). Nail samples (patient IV-2) and a palmoplantar swab (patient IV-5) were positive for *Trichophyton rubrum*.

A skin biopsy of patient III-2 (axillae) showed histologically normal number and aspect of eccrine sweat glands (ESG).

**Figure 2:**
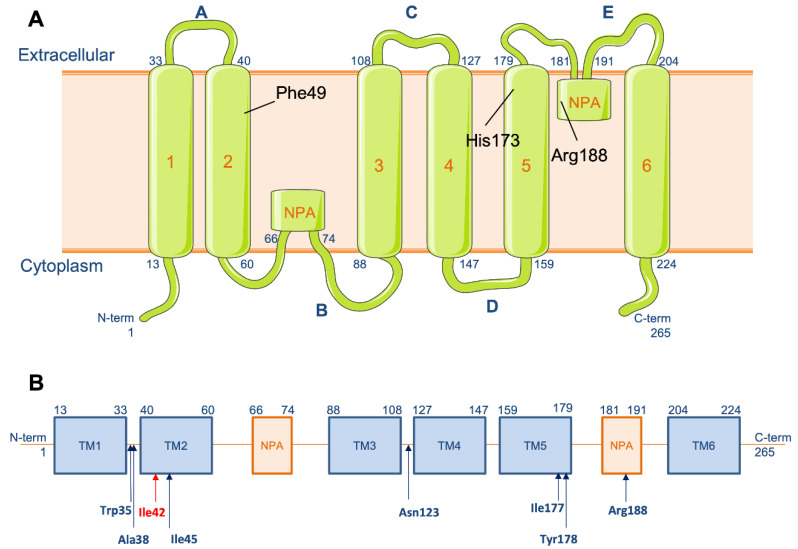
**Aquaporin 5 protein and *AQP5* gene.** (**A**) Schematic representation of the aquaporin-5 protein; (**B**) localization of the mutation c.125T>A p.(Ile42Asn in red) and previously reported mutations on *AQP5* gene in blue.

Using next-generation sequencing of a targeted panel of genes involved in genodermatoses, a heterozygous missense mutation (c.125T>A; p.(Ile42Asn); Figure 2) in *AQP5* was identified. This variant was absent from all public databases (including gnomAD, dbSNP, 1000 Genomes), and it segregated with the disease.

### 3.2. Sugar and Sweat Tests

Complete dissolution of the 5.95 g sugar cube was obtained at 2′17 (III-2), 3′03 (IV-2), 1′43 (IV-3) and 2′00 (IV-5). Salivary secretion rate was similar to controls.

Sweat Cl^−^ concentrations were within the normal range for three patients: IV-2 (20 mmol/L), IV-5 (16 mmol/L) and IV-7 (21 mmol/L), and in the intermediate range for two patients: III-2 (40 mmol/L) and IV-3 (31 mmol/L).

## 4. Discussion

The PPKB subtype is a rare autosomal dominant inherited PPK. We report four French patients belonging to the same family and carrying a novel *AQP5* heterozygous mutation, and refine the associated phenotype.

In PPKB, palmoplantar swelling is reported to be the first manifestation, starting after one year of age [1]. We showed that palmoplantar swelling might start from birth (patients III-2, IV-2 and IV-5) or occur during the first year of life (patient IV-7). White spongy swelling of the stratum corneum or “hand-in-the-bucket sign” is also induced after 1 to 15 min of water immersion [3,12,13,14] or after excessive sweating. While swelling was significantly evident after 6 min of immersion for the father, they occurred earlier, after 3 min of immersion, in the children. Interestingly, pruritus and hyperhidrosis with unpleasant odor were mentioned by all four patients. They represent an early and constant manifestation, reported in 27/28 families (Table 1).

Slight focal hyperkeratosis of the palms and soles, including fingers and toes, starts as early as six months (patient IV-7). With age, the yellowish PPK extended and thickness worsened becoming pigmented, with a papular and inflammatory border. Progressively, the PPK becomes transgrediens, non-progrediens, homogeneous and diffuse with a clear delineation between normal and affected skin (patient III-2, Figure 1B). The transgrediens characteristic remains inconstant. However, published pictures frequently show extension on the dorsum of the toes [2,12]. UV light (Woods lamp) examination highlights the affected skin areas and can show *Corynebacterium* infection [2].

PPKB should be distinguished from the acquired aquagenic PPK such as HPA and AWP. In both, manifestations are induced by water immersion of normal skin. HPA associates fine-textured scalp hair and atopic diathesis [6] and persistent, asymptomatic, yellowish-white, translucent papules and plaques on the hands and feet that exacerbate after water exposure. AWP is characterized by translucent papules on the palms that frequently spares soles [7].

The *AQP5* gene encodes a 265 amino-acid monomer characterized by six trans-membrane domains, and two hydrophobic asparagine-proline-alanine sequences (NPA), belonging to a large group of at least 13 human channels responsible for rapid bidirectional osmotic water movement across the plasma membrane in many cell types. The *AQP5* gene is expressed in ESG, salivary and airway submucosal glands [15]. Hyposialia (sugar test), and hypolacrimia were never reported. In our patient, the skin biopsy was taken in the axillae to explore the aspect of ESG, which appear normal under light microscopy. Probably due to ESG density, the PPKB phenotype seems to be limited to palms and soles.

During sweating, AQP5 rapidly translocates to the apical membrane, increasing the water permeability of ESG [16]. Water flow moves in both directions through the channels formed by each of the four AQP5 monomers rather than through the central pore of the tetramer. Two NPA motifs localize closely to form two hemipores facing each other in reverse, closely restricting water passage. Arg188, Phe49 and His173 define the constriction point of the channel [3].

In a recent Finnish study exploring mutations underlying hereditary PPK in 64 patients, the heterozygous *AQP5* c.113C>A p.Ala38Glu mutation was the most common finding overall and in patients with diffuse PPK [17]. The majority of the reported mutations, including that of our family, are located on the N-terminal extremity (n = 22/28) and, especially, on the extracellular surface of AQP5 (n = 19/28). More generally, the mutations line the extracellular end of the water channel (Ile42, Ile45, Ile177, Tyr178 and Arg188) when they are not located on the extracellular surface (Trp35, Ala38 and Asn123) (Figure 2). Considering the tertiary structure of the protein, these regions could play a key role in water conductance. On the other hand, the role of the C-terminal domain has been reported as crucial for its trafficking to the plasma membrane [18]. In PPKB, gain of function mutations seem to widen the channel diameter [3] and increase water movement. Therefore, swelling is induced by hypotonicity with water entrance into cells, while hyperhidrosis is the result of increased cytosolic calcium concentration [13]. While sweat Cl^−^ concentrations were normal in three of four of our PPKB patients, further sweat analyses, i.e., electrolytes and proteins [19], will highlight the key role of the ESG in the PPKB pathophysiology.

## Figures and Tables

**Table 1:** **PPKB: Clinical and molecular characterization of the previously reported families (F1–27) and the present report (F28)**. NA, not available. Age and gender were not specified in families F1 to F27.

Families	Total of Families/Patients	Background	Clinical Manifestations	Water–Immersion Test	Mutation	Protein
F1 [1]	1/NA	British	Diffuse, yellowish-white, transgradient nonepidermolytic palmoplantar keratoderma affecting palms and plants. Variability of expression. Hyperhidrosis.	In 15 min, white spongy appearance	c.529A>T	p.Ile177Phe
F2–3 [1]	2/NA	British			c.134T>G	p.Ile45Ser
F4–10 [1]	7/NA	Swedish			c.113C>A	p.Ala38Glu
F10 [1]	1/NA (Among the 7)	Swedish			c.562C>T	p.Arg188Cys
F11 [1]	1/NA	Scottish			c.367A>G	p.Asn123Asp
F12 [2]	1/14	Danish	Diffuse, yellowish-white, nonepidermolytic palmoplantar keratoderma, affecting palms and plants. Non- transgradient. Hyperhidrosis.	In 3 min, translucent white papules and whitish spongy appearance	c.562C>T	p.Arg188Cys
F13 [3]	1/8	Chinese	Diffuse, homogenous, palmoplantar hyperkeratosis, which extended to the lines of transgredience with an erythrokeratotic margin, affecting palms and plants Non- transgradient. Hyperhidrosis.	In 1 min, whitish spongy appearance	c.533A>G	p.Tyr178Cys
F14 [3]	1/6	Chinese			c.367A>T	p.Asn123Tyr
F15 [4]	1/10	Chinese	Diffuse, yellowish-white, mild, transgradient, progressive thickening erythrokeratotic plaques with a clear demarcation on the dorsum of hands and feet, affecting palms and plants. Hyperhidrosis.	In 1 min, whitish spongy appearance	c.367A>T	p.Asn123Tyr
F16 [5]	1/3	Japanese	Mild, diffuse, transgradient, palmar erythema with scales arranged along or around palmar and finger folds.	Palm skin wrinkled quickly	c.104G>C	p.Trp35Ser
F17–27 [6]	11/11	Finnish	Mild, diffuse, palmoplantar keratoderma. Secondary dermatophyte infections. Hyperhidrosis.	Aquagenic whitening	c.113C>A	p.Ala38Glu
F28	1/4	French	Diffuse, yellowish-colored, transgradient, palmoplantar hyperkeratosis. Worsening of symptoms over time. Hyperhidrosis.	In 6 min, whitish spongy appearance	c.125T>A	p.Ile42Asn

## Data Availability

All the data are published in the present article.

## References

[1] Lind L., Lundström A., Hofer P.A., Holmgren G. (1994). The gene for diffuse palmoplantar keratoderma of the type found in northern Sweden is localized to chromosome 12q11-q13. Hum. Mol. Genet..

[2] Krøigård A.B., Hetland L.E., Clemmensen O., Blaydon D.C., Hertz J.M., Bygum A. (2016). The first Danish family reported with an AQP5 mutation presenting diffuse non-epidermolytic palmoplantar keratoderma of Bothnian type, hyperhidrosis and frequent Corynebacterium infections: A case report. BMC Dermatol..

[3] Blaydon D., Lind L., Plagnol V., Linton K., Smith F.D., Wilson N., McLean W.H., Munro C., South A., Leigh I. (2013). Mutations in AQP5, encoding a water-channel protein, cause autosomal-dominant diffuse nonepidermolytic palmoplantar keratoderma. Am. J. Hum. Genet..

[4] Nielsen P.G. (1988). Hereditary palmoplantar keratoderma and dermatophytosis. Int. J. Dermatol..

[5] Elmros T., Lidén S. (1981). Hereditary palmo-plantar keratoderma: Incidence of dermatophyte infections and the results of topical treatment with retinoic acid. Acta Derm. Venereol..

[6] Köster W., Nasemann T. (1985). Hereditary papulotranslucent acrokeratoderma. Z. Hautkr..

[7] English J.C., McCollough M.L. (1996). Transient reactive papulotranslucent acrokeratoderma. J. Am. Acad. Dermatol..

[8] Kaiser H., Brustad N., Pressler T., Bygum A. (2018). Aquagenic wrinkling of the palms in patients with cystic fibrosis. Br. J. Dermatol..

[9] Gild R., Clay C.D., Morey S. (2010). Aquagenic wrinkling of the palms in cystic fibrosis and the cystic fibrosis carrier state: A case-control study. Br. J. Dermatol..

[10] Wolff A., Herscovici D., Rosenberg M. (2002). A simple technique for the determination of salivary gland hypofunction. Oral Surg. Oral Med. Oral Pathol. Oral Radiol. Endodontol..

[11] Cirilli N., Southern K.W., Barben J., Vermeulen F., Munck A., Wilschanski M., Nguyen-Khoa T., Aralica M., Simmonds N.J., de Wachter E. (2022). Standards of care guidance for sweat testing; phase two of the ECFS quality improvement programme. J. Cyst. Fibros..

[12] Pan Y., Men Y., Lin Z. (2019). Palmoplantar keratoderma Bothnia type with acrokeratoelastoidosis-like features due to AQP5 mutations. Clin. Exp. Dermatol..

[13] Cao X., Yin J., Wang H., Zhao J., Zhang J., Dai L., Zhang J., Jiang H., Lin Z., Yang Y. (2014). Mutation in AQP5, encoding aquaporin 5, causes palmoplantar keratoderma Bothnia type. J. Investig. Dermatol..

[14] Wada Y., Kusakabe M., Nagai M., Imai Y., Yamanishi K. (2019). Japanese case of Bothnian-type palmoplantar keratoderma with a novel missense mutation of p.Trp35Ser in extracellular loop A of aquaporin-5. J. Dermatol..

[15] Verkman A.S. (2011). Aquaporins at a glance. J. Cell Sci..

[16] Inoue R., Sohara E., Rai T., Satoh T., Yokozeki H., Sasaki S., Uchida S. (2013). Immunolocalization and translocation of aquaporin-5 water channel in sweat glands. J. Dermatol. Sci..

[17] Harjama L., Karvonen V., Kettunen K., Elomaa O., Einarsdottir E., Heikkilä H., Kivirikko S., Ellonen P., Saarela J., Ranki A. (2021). Hereditary palmoplantar keratoderma—phenotypes and mutations in 64 patients. J. Eur. Acad. Dermatol. Venereol. JEADV.

[18] Muroi S.-I., Isohama Y. (2021). C-terminal Domain of Aquaporin-5 Is Required to Pass Its Protein Quality Control and Ensure Its Trafficking to Plasma Membrane. Int. J. Mol. Sci..

[19] Cornet M., Nguyen-Khoa T., Kelly-Aubert M., Jung V., Chedevergne F., le Bourgeois M., Aoust L., Roger K., Guerrera C.I., Sermet-Gaudelus I. (2022). Proteomic profiling of sweat in patients with cystic fibrosis provides new insights into epidermal homoeostasis. Ski. Health Dis..

